# A Query-Based Network for Rural Homestead Extraction from VHR Remote Sensing Images

**DOI:** 10.3390/s23073643

**Published:** 2023-03-31

**Authors:** Ren Wei, Beilei Fan, Yuting Wang, Rongchao Yang

**Affiliations:** 1 Institute of Agricultural Information, Chinese Academy of Agricultural Sciences, Beijing 100876, China; 2 Key Laboratory of Agricultural Blockchain Application, Ministry of Agriculture and Rural Affairs, Beijing 100125, China

**Keywords:** VHR remote sensing images, rural homestead, instance segmentation, query-based, multi-scale deformable attention, group queries

## Abstract

It is very significant for rural planning to accurately count the number and area of rural homesteads by means of automation. The development of deep learning makes it possible to achieve this goal. At present, many effective works have been conducted to extract building objects from VHR images using semantic segmentation technology, but they do not extract instance objects and do not work for densely distributed and overlapping rural homesteads. Most of the existing mainstream instance segmentation frameworks are based on the top-down structure. The model is complex and requires a large number of manually set thresholds. In order to solve the above difficult problems, we designed a simple query-based instance segmentation framework, QueryFormer, which includes an encoder and a decoder. A multi-scale deformable attention mechanism is incorporated into the encoder, resulting in significant computational savings, while also achieving effective results. In the decoder, we designed multiple groups, and used a Many-to-One label assignment method to make the image feature region be queried faster. Experiments show that our method achieves better performance (52.8AP) than the other most advanced models (+0.8AP) in the task of extracting rural homesteads in dense regions. This study shows that query-based instance segmentation framework has strong application potential in remote sensing images.

## 1. Introduction

As an important part of China’s rural areas, rural homesteads are directly related to the life and economic development of farmers. In recent years, the Chinese government has begun to gradually attach importance to the management of rural homesteads, and comprehensively grasp the relevant data of rural homesteads through an information survey. With the rapid development of satellites, unmanned aerial vehicles, and other aerospace platforms, a wide range of very high-resolution (VHR) remote sensing images can be obtained. At present, VHR images have been used in a large number of fields, such as cadastral mapping, land classification, road extraction, and rural planning and change detection [1,2,3,4]. VHR images can help us analyze the target objects clearly and accurately. Combined with the advantages of VHR imaging, the current survey method of information such as the quantity and area of homestead has changed from traditional field mapping to manual remote sensing image interpretation, and the rural homestead map spots are extracted by visual interpretation. Although high-resolution remote sensing images can provide clear and large-scale ground information, the distribution characteristics of rural homesteads are dense and large in number. The cost of manually extracting homestead map spots from remote sensing images is high and the efficiency is low, thereby creating difficulties to meet the national demand for large-scale homestead information statistics. Therefore, a fast and accurate automation method is needed to solve this problem.

A remote sensing image has the characteristics of multi-scale, diversity, light sensitivity, complex features, and so on [5]. Making full use of remote sensing image features is the key factor to improve the performance of recognition methods. Some traditional methods achieve the target extraction task by manually extracting morphological indicators, spectral features, and some semantic features of remote sensing images [6,7,8]. However, these indicators are sometimes unreliable. Changes in the shooting angle, illumination conditions, intra-class spectral changes, and inter-class spectral similarity of remote sensing images often limit the application of these methods [9]. AlexNet won the championship in the ImageNet competition in 2012 [10]. After that, deep learning began to show its great potential in the field of images and gradually entered the vision of researchers. People try to apply depth learning technology to image object detection and segmentation, and have achieved great success [11,12]. Compared with traditional algorithms, deep learning does not need to rely on manually extracted prior knowledge and can automatically learn multi-level and multi-dimensional implicit information, which provides an efficient and stable method for interpreting remote sensing images with rich surface feature information. Before 2020, the field of computer vision mainly used CNN as the main framework of deep learning. Here, by stacking convolutional blocks with convolutional kernels of different sizes, the receptive field is gradually increased to extract low-level to high-level semantic information. Many excellent instance segmentation algorithms based on CNN architecture have been proposed. These algorithms are often divided into two methods: top-down and bottom-up. The top-down method is generally to perform target detection first and then segmentation, whereas the bottom-up method is generally to perform semantic segmentation first, and then distinguish different instances through clustering or other measurement methods. However, the post-processing steps of the bottom-up method are often cumbersome, and the accuracy is not high. All the current mainstream methods are top-down methods. The top-down method is divided into the one-stage method and two-stage method according to whether the target detection network needs a proposal region. The two-stage method requires that the candidate boxes are classified, regressed, and masked. Such methods are represented by Mask-RCNN, Cascade-RCNN, HTC, etc. [13,14,15,16]. They can often achieve better accuracy, but the computational cost is large, and the speed is slow. The one-stage method does not need to generate a proposal region, but directly predicts the category, location, and mask of the target. This type of method represents YOLACT, BlendMask, SOLOV1, SOLOV2, etc. [17,18,19,20]. The advantages of this type of method are high efficiency, low computational overhead, but the accuracy cannot be guaranteed. These two types of methods have a common problem. They depend on NMS-related operations, which makes them unable to become end-to-end network architectures. Vision Transformer (VIT), proposed in 2020, is the first model to successfully apply transformer architecture to the field of computer vision [21]. Subsequently, a transformer-based object detection algorithm Detection Transformer (DETR) was proposed [22]. It is the first end-to-end network in the true sense, and it does not need to manually set a threshold to filter out reasonable target boxes and masks. DETR uses a new idea to detect objects, and searches for instance regions in images by querying. This architecture is simpler and more elegant. At the same time, the emergence of DETR also opens a new idea for instance segmentation. Instance segmentation does not need the traditional top-down method of detection and segmentation but can also be completed by finding the mask of the instance region on the image, which is called the query-based method. QueryInst, MaskFormer, Mask2Former, and other advanced instance segmentation are also designed based on the query-based method [23,24,25,26]. This kind of method has high accuracy and is simpler in structure than the traditional top-down instance segmentation method. Moreover, Transformer’s attention mechanism has the ability to capture global context information, so it is more suitable for remote sensing images that have rich context semantic information and complex scenes.

**Figure 1:**
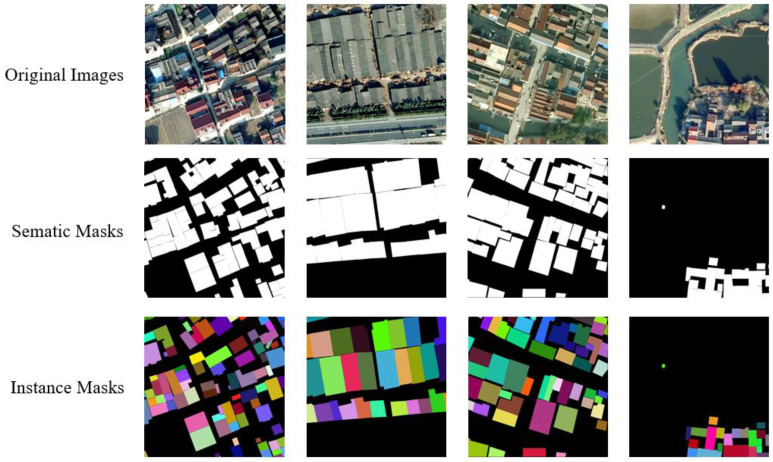
Semantic labels and instance labels of rural homestead in dense areas.

Although the current instance segmentation method has achieved excellent performance, it is rarely used in remote sensing images. Fang et al. designed an attention module and a boundary optimization module to improve the detection performance of small target buildings [27]. Li et al. used the Mask-RCNN framework to identify new and old buildings in rural areas [28]. Wu et al. proposed an Anchor free framework to solve the problem of building instance segmentation [29]. Liu et al. uses an improved HTC algorithm to solve the problem of building instance segmentation [30]. Although these CNN-based methods have good performance, the overall structure is relatively complex, and end-to-end building instance segmentation cannot be achieved. A large number of manually set thresholds, such as anchor’s aspect ratio, confidence, and NMS threshold are required. When buildings show significant shape differences and scale variability, some anchor-free detectors cannot accurately regress the position caused by fixed grid calculation in convolution [9]. Most remote sensing image interpretation tasks, especially building extraction tasks, are based on semantic segmentation models, and most remote sensing image building extraction dataset labels are also designed manually for semantic segmentation [31,32,33,34]. Although the performance of the semantic segmentation model is already well, it can not achieve satisfactory results when encountering very dense and continuous regions in rural areas. As shown in Figure 1, the distribution of buildings in rural areas in China tends to show an aggregation pattern, scattered in different locations in the form of villages, and the distribution of rural homesteads in villages is very dense. It is easy to mistakenly identify two closely connected rural homesteads as one, which will lead to errors in subsequent statistical information. Although there are rare related works using semantic segmentation models to separate adjacent rural homesteads [35], when there is overlap in the space of adjacent rural homesteads, the semantic labels only mark them as the category of homesteads. However, it is impossible to distinguish instance individuals, which has caused huge interference to the statistical quantity and area.

To solve the above problems, we propose a network for rural homestead extraction in very dense areas based on the simpler query-based architecture. The main contributions of this study are summarized as follows:

● We propose an end-to-end instance segmentation method based on Transformer architecture and build a multi-scale deformable attention module in encoder to aggregate cross hierarchical global context information to enhance distance dependency, in addition, this greatly reduces the number of parameters and improved the convergence speed;
● Group queries are built in the decoder, which changes the One-to-One label assignment method of the original DETR to the Many-to-One label assignment method, greatly improving the convergence speed of the decoder. Moreover, no additional parameters are required in the inference stage;
● We propose a method to solve the problem of it being difficult to accurately extract rural homestead due to its dense distribution. The extracted pattern can be used to count the number, area, and other information regarding homesteads.

## 2. Materials and Methods

### 2.1. Data Collecting

Our data were collected from UAV images with a spatial resolution of 0.2 m, covering the whole area of Deqing County, Zhejiang Province, China. We used the LabelMe annotation tool to manually mark rural homesteads in a partial cropping area and generate binary mask label images. To generate instance-level masks, we also saved the JSON files generated by the LabelMe tool.

### 2.2. Methods

#### 2.2.1. Overview of Network Architecture

**Figure 2:**
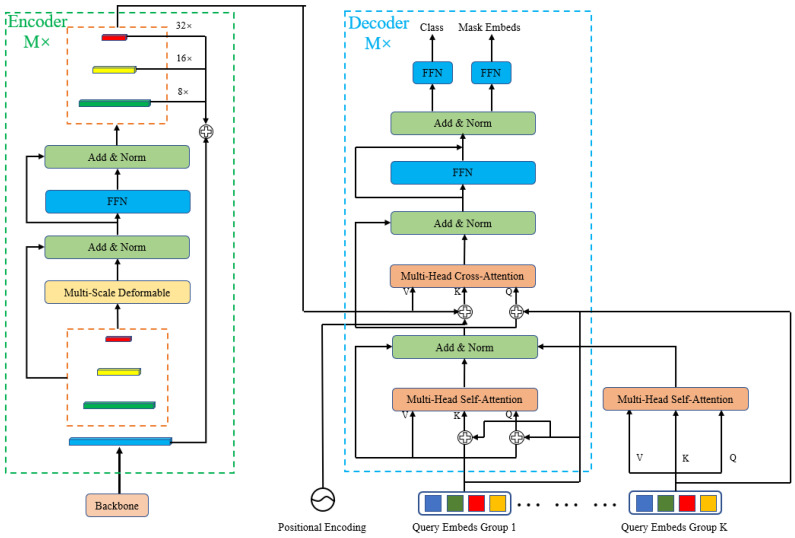
Architecture of QueryFormer.

As shown in Figure 2, the QueryFormer network proposed in this paper is based on Transformer architecture. Aggregation of context information is important for remote sensing images with multi-scale information. The Transformer architecture is spatially capable of long-distance modeling, captures context-dependent, long-distance relationships, and is well suited for remote sensing imagery. In QueryFormer, there is an encoder and a decoder. To extract features, we utilize the Swin-Transformer network as the backbone, which has recently demonstrated excellent performance [36]. The Swin-Transformer network addresses the limitations of the original Vision Transformer, which struggles to extract multiscale information and is not well-suited for small targets in images. After extracting the information using Swin-Transformer, the feature maps with four resolutions are obtained, which are 1/4, 1/8, 1/16, and 1/32. High-resolution feature maps can extract underlying information such as image boundaries and textures, and are friendly to small targets, whereas low-resolution images have strong semantic features. Feature maps with 1/8, 1/16, and 1/32 down-sampling resolution were input into the Multi-Scale Deformable Attention module (see Section 2.2.2) to obtain a self-attention feature map. In order to obtain high-quality mask feature maps, the self-attention feature maps of three resolutions were up sampled to the highest resolution feature map and added together to obtain high-resolution feature maps with rich boundary and semantic information. QueryFormer decoder is similar to DETR decoder, and we first initialize group queries embedding (see Section 2.2.4), which is shaped as K × N × C, where K is the number of groups, N is the number of queries, and C is the number of embedding dimensions. In this experiment, we set K to 5 and N to 300. In the decoder, the standard self-attention module and cross-attention module (see Section 2.2.3) are used to compute from multi-scale image features and N learnable positional embeddings from its output. Each mask query predicted by QueryFormer is encoded using *N* per-mask embeddings $Q\in R^{C_{Q}\times N}$ of dimension C_Q_, which capture global information. The query results are then passed through a linear classifier layer (FFN layer) to generate decoder embeddings. These decoder embeddings are further processed by an MLP layer and a SoftMax activation layer to produce class probability embeddings and mask embeddings, respectively (as described in Section 2.2.4).

#### 2.2.2. Multi-Scale Deformable Attention Module

In order to enhance the effect of feature extraction and aggregate context information, we add an attention mechanism behind the backbone. At present, the mainstream methods to solve the task of target detection and instance segmentation are a process from dense to sparse. They all need dense prior knowledge (anchor, reference points, etc.), but there are some problems with dense prior. Firstly, many similar results will be detected, and post-processing methods such as threshold and NMS are required to filter. Secondly, the detection results are closely related to a priori (the number and size of anchors, the degree of confidentiality of reference points, the number of proposals generated, etc.). These super parameters need to be carefully initialized. Carion et al. proposed a novel object detection model, called DETR, which employs an encoder–decoder structure combined with the powerful global modeling capability of the Transformer architecture [22]. By eliminating the need for manual design of anchors, reference points, and other prior knowledge, as well as post-processing techniques such as NMS, they were able to create the first completely end-to-end object detector. Although the DETR has better performance and simpler ideas, there are still some problems. Firstly, compared with the mainstream object detectors, the DETR needs more time to train to converge. Secondly, the effect of DETR on small target detection is not effective. The main reasons for the above problems are as follows. Firstly, the Transformer structure has defects in processing image feature maps. During initialization, the attention module applies nearly consistent attention weights to all pixels in the feature map. A long training time is necessary to focus on a sparse and meaningful position. On the other hand, the calculation of the weight of attention in the Transformer encoder is a secondary calculation of the number of pixels. Therefore, processing high-resolution feature maps has very high computational and memory complexity. Secondly, target detectors with good performance use multi-scale features to detect small objects from high-resolution feature maps, which make DETR highly complex and unacceptable. In order to solve the above problems, encouraged by Deformable DETR [37], we designed a multi-scale Deformable attention (MSDeformattn) module in the network. The MSDeformattn module combines Deformable Evolution’s sparse feature space sampling and Transformer’s global modeling capabilities to alleviate the slow convergence and high complexity of the DETR algorithm.

**Figure 3:**
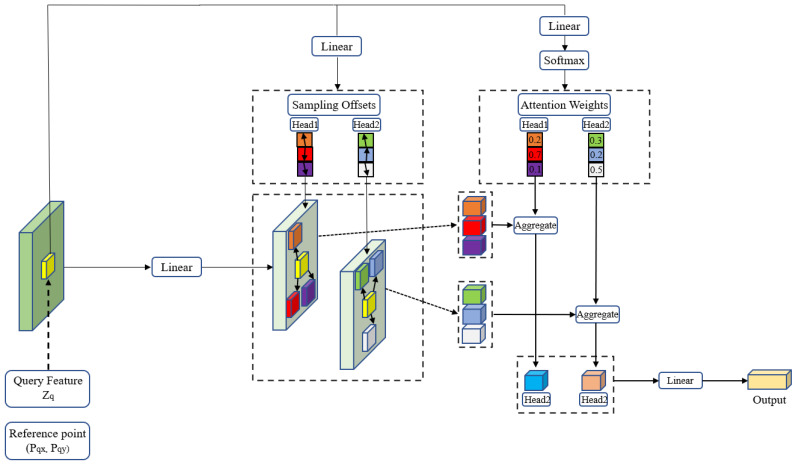
The structure of the single scale deformable attention module.

Give a scale feature map $x\in R^{C\times H\times W}$, the expression of deformable attention is:

(1) $$DeformAttn\left(z_{q},p_{q,}x\right)=\sum_{m=1}^{M}W_{m}\left[\sum_{k=1}^{K}A_{mqk}\cdot W_{m}{}^{\prime }x\left(p_{q}+\Delta p_{mqk}\right)\right]$$

where *q* is the index of a feature location on the feature map *x*, *z_q_* is the corresponding feature vector on *x* for the index *q*, and *p_q_* is the corresponding two-dimensional reference point on *x* for the index *q*. *M* represents the number of attention heads, *K* represents the number of sampled keys, *W* and $W^{\prime }$ represent two different linear projection layers. *A_mqk_* denotes attention weights at the *k*th sampling point of the *m*th attention head corresponding to index *q*, *p_mqk_* denotes learnable sampling offsets at the *k*th sampling point of the *m*th attention head corresponding to index *q*, and $x\left(p_{q}+\Delta p_{mqk}\right)$ is calculated by bilinear interpolation. Figure 3 shows that there are two attention heads (*M* = 2) and three sampling offset points (*K* = 3) on a single scale. First, flatten the single scale feature map $x\in R^{C\times H\times W}$ shape to C×N, where N=H×W and *C* is the number of original channels. Unlike the self-attention module in the Vision Transformer, the query feature is not generated by linear projection of the original sequence, but the original sequence with position encoding. Then the query feature is passed through a linear projection layer with an output channel of 2*MK* to obtain the encoded sampling offsets $\Delta p_{mqk}$. For generating the encoded attention weights $A_{mqk}\in \left(0,1\right)$, the query feature is passed through another linear projection layer with output channel of *MK* and SoftMax activation layer. Multiply $A_{mqk}$ with the characteristic value of a resampled offset point through the linear projection layer to obtain the feature map after deformable attention.

The multi-scale deformable attention module is to sample *LK* points on the feature map of multiple levels, then perform the above operations separately. Finally, the feature maps of all scales are added together to get the multiscale feature map, which is expressed as

(2) $$MSDeformAttn\left(z_{q},{\overset{\wedge }{p}}_{q,}{\left\{x^{l}\right\}}_{l=1}^{L}\right)=\sum_{m=1}^{M}W_{m}\left[\sum_{l=1}^{L}\sum_{k=1}^{K}A_{mlqk}\cdot W_{m}{}^{\prime }x^{l}\left(\phi_{l}\left({\overset{\wedge }{p}}_{q}\right)+\Delta p_{mlqk}\right)\right]$$

where *L* denotes *L* the number of levels, and other parameters is the same as the parameters in Expression (2).

#### 2.2.3. Self-Attention Module and Cross-Attention Module

**Figure 4:**
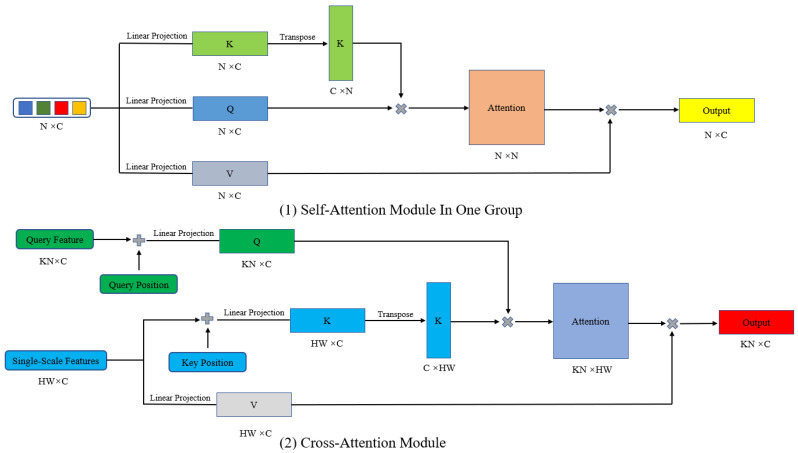
Self-Attention Module In One Group and Cross-Attention Module.

Inspired by DETR structure, we add two attention modules to decoder, which are self-attention module and cross-attention module. Its structure is shown in Figure 4. Their expressions are shown in Equations (3) and (4).

(3) $$Self-Attention\left(Q_{s},K_{s},V_{s}\right)=soft\max\left(\frac{Q_{s}K_{s}{}^{T}}{\sqrt{d_{k}}}\right)V_{s}$$

(4) $$Cross-Attention\left(Q_{c},K_{c},V_{c}\right)=soft\max\left(\frac{Q_{c}K_{c}{}^{T}}{\sqrt{d_{k}}}\right)V_{c}$$

First, query embeds in each group initialized to *N* × *C* are divided into three same parts: query, key, and value. Their shapes are also *N* × *C*. Query and key are added with the same query position embeds, and then they are separately passed through a linear projection layer to obtain *Q_s_*, *K_s_*. Enter value into a linear projection layer to get *V_s_*. *K* is transposed to a sequence with a shape of C×N, multiplied by *Q*, attention weights are obtained through the SoftMax activation function, and *V* and attention weights are multiplied to get an output sequence with a shape of N×N. By adding query embeds and outputs and passing through the LayerNorm layer, the weighted query feature is obtained, and the self-attention operation is completed. Following this is the cross-attention module, which adds query position embedding to the query feature obtained from the self-attention module and obtains Qc∈KN×C through a linear projection layer, whereas the key and value in the cross-attention come from the three-layer feature map in the encoder. Kc∈HW×C is obtained by adding key position embedding and passing through a linear projection layer. $V_{C}\in HW\times C$ is formed by value passing directly through a linear projection layer. The steps to obtain attention weights and features follow the same pattern as self-attention. The result’s shape is *KN* × *C* after cross-attention module.

#### 2.2.4. Group Queries Assignment

**Figure 5:**
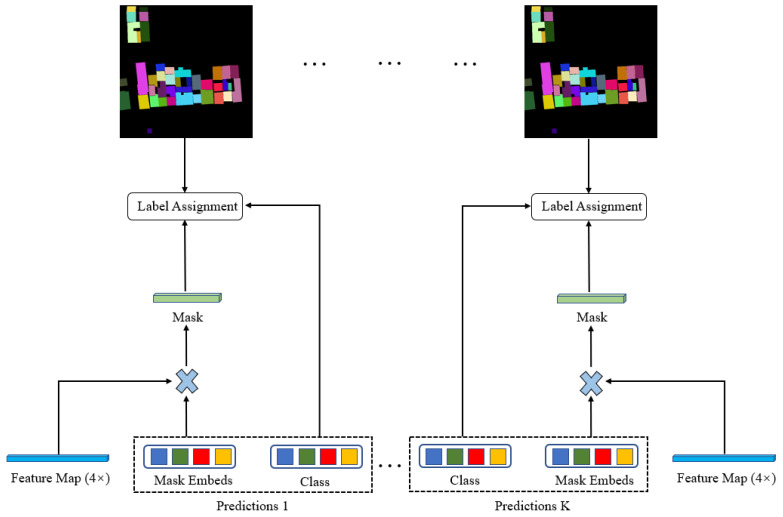
Group queries assignment in training stage.

In the traditional CNN-based object detection algorithm, a feature region is usually mapped by multiple proposal boxes, and then the prediction box closest to the label is obtained through NMS or other post-processing methods. This is equivalent to a target instance being predicted by multiple anchors, which can focus on the target region more quickly and is a Many-to-One matching method. The end-to-end method DETR discards the NMS operation, but it cannot perform Many-to-One label matching, which makes the convergence speed very slow. Therefore, we use multiple groups of queries to perform many to one matching. This matching method only occurs in the training process, and in the inference stage, it is only necessary to take the first group of queries for forward propagation, so that the calculation consumption during reasoning is not increased. As shown in Figure 5, there are K groups of queries. In the forward propagation process, K groups of predictions are obtained. Each group of predictions consists of category probability prediction vectors and mask embeds. The mask embeds shape is N × C, it multiplies the high-resolution feature map with the shape of C × 4/H × 4/W in encoder to obtain the shape of N × 4/H × 4/W mask prediction. Each group of prediction matches the label in the One-to-One way, and then updates the weight through the loss function. In this way, a region in the label is mapped by the feature regions extracted by K queries, so as to achieve the goal of many to one matching.

#### 2.2.5. Loss Design

As a network architecture for end-to-end instance segmentation, QueryFormer’s loss function consists of two parts: classification loss and mask loss. The classification loss uses focal loss, whereas the mask loss uses the weighted sum of cross entropy loss and dice loss [38].

For remote sensing images, there is often a large number of unbalanced categories, so focal loss is used to deal with the unbalance distribution of categories. The calculation formula of focal loss is shown in Equation (5).

(5) $$FL\left(p_{t}\right)=-\partial_{t}{\left(1-p_{t}\right)}^{\gamma }\log\left(p_{t}\right)$$

where, $\partial_{t}\in \left(0,1\right)$ is a super parameter used to control the imbalance of positive and negative samples, $\gamma \in \left(0,5\right)$ is a super parameter to balance difficult samples and simple samples, and $p_{t}$ is the probability distribution of prediction of different categories. For positive samples, when P is large, the category confidence of prediction is high, and it is a simple sample, so ${\left(1-p_{t}\right)}^{\gamma }$ is small, and the loss value is small. On the contrary, when p is small, the sample confidence is low, and the loss value is large. For negative samples, when P is small, the prediction category is more inclined to negative samples, and the loss value is small. On the contrary, when P is large, the loss value is large. By adjusting γ to reduce the impact of simple samples, the network is made pay more attention to difficult samples. QueryFormer outputs a mask shape of B×Q×H×W at the last layer of decoder in one group (noted that we only introduce one group loss here, and is same as other group), where B is batch size, Q is the number of queries, and H and W are the height and width of a quarter of the input image. For most segmentation networks, the output shape is B×C×H×W, C is the classes numbers, and the quantity of Q is much larger than the quantity of C. Using the mask result which shape is B×Q×H×W to calculate the loss will result in enormous training memory consumption. Inspired by some efficient work, we calculate the loss function by selecting K sample points for each H×W feature map based on the uncertainty of the current pixel instead of the entire feature map [16,25], where the uncertainty is measured based on the pixel prediction confidence. The formula for calculating classification loss is shown in Equation (5).

## 3. Results

### 3.1. Dataset

The original image datasets were labeled by LabelMe to general semantic masks, JSON file was used to assign an instance to each mask object, and then we generated the instance masks. Then, we produced the COCO format annotation JSON files. The semantic masks generated from LabelMe tool, and the instance masks generated by converting are shown in Figure 5 below. The size of each image data used for training and testing is more than 10,000 × 10,000, which cannot be put into the GPU devices for calculation. Therefore, we use the overlapping clipping method to cut out several 640 ∗ 640 images, with the size of the middle overlapping part of 200. If the length remaining after cropping the last image of a row or column is less than 640, the final image of the row or column will be cropped from its size minus 640 to its original size. Finally, the number of cut sample data used for training is 22,338, and the number of data used for testing is 3630. In the training stage, we use data augmentation methods such as random flipping, random scaling and clipping, and random color change for cropped images.

### 3.2. Experimental Environment and Details

The experimental environment is based on the Ubuntu 18.04 system, using Python 3.8 and PyTorch 1.12.1 to build models and train. The hardware conditions are Intel (R) Xeon (R) CPU E5-2678 v3 @ 2.50G Hz, 256G RAM, and 8 × 24G RTX3090 GPUs. At the beginning of training, we set the initial learning rate to 0.0001, and use warmup strategy to increase the learning rate [39]. After 10,000 steps, we used the poly method to decrease learning rate [40]. The poly method is shown in Equation (6).

(6) $$lr=base\_lr\times {\left(1-\frac{cur\_iters}{\max\_iters}\right)}^{power}$$

where base_lr is the last learning rate, and cur_iters is the current number of steps, max_iters is the total number of training steps, and the value of power is set to 0.9. The optimizer we chose to update the model’s weights is AdamW [41]. We set the batch size per GPU is 2, and the total batch size is 16.

### 3.3. Comparison of Experimental Results

In order to prove the effectiveness of our proposed network, we compared the results with those of other SOTA models under the same experimental conditions, including Mask-RCNN, Cascade-RCNN, QueryInst, and Mask2Former. The first three are instance segmentation models based on CNN architecture, and they follow the top-down detection paradigm. Moreso, the last two are models based on the Transformer architecture and follow the query method to obtain the prospect target. All methods adopt ResNet [42] and Swin-Transformer as the backbone, and we selected ResNet101 and Swin-B for comparison. To make a fair comparison, we list the methods in which different algorithms query instances. The CNN-based method uses anchor boxes, whereas the Transformer-based method uses 300 randomly initialized learnable queries (QueryFormer uses five groups for training and one group for inferencing). Table 1 shows the performance comparison results of QueryFormer with other SOTA methods. In contrast experiments, we only evaluate the quality of the instance mask. The integrity of the Transformer-based method is better than that of the CNN-based method. In the experiment with Swin-B as the backbone, the average mask precision of our QueryFormer was 52.8%, which is 0.8% higher than Mask2Former and 1.7% higher than HTC. The average precision of medium-sized targets (AP_M_) was 1.0%, higher than Mask2Former and 2.1% higher than HTC. The average precision of large targets (AP_L_) was 0.9% higher than Mask2Former and 2.4% higher than HTC. In the comparison of indicators AP_S,_ which are used to evaluate the segmentation quantitative of small targets, the highest one was HTC, and QueryFormer was 0.2% lower than QueryInst.

**Figure 6:**
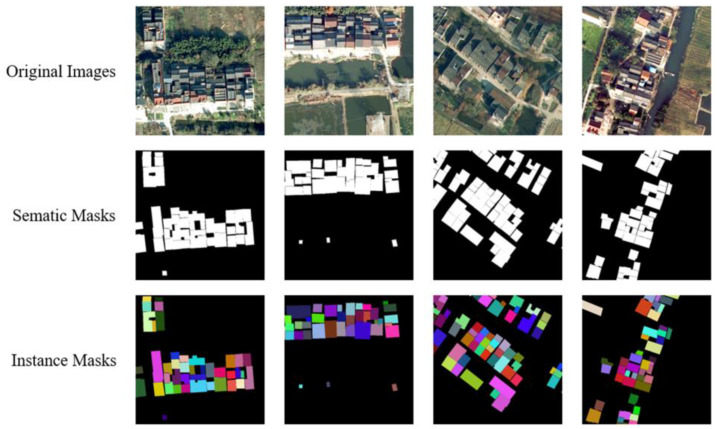
Examples of training and testing data sets.

In order to better compare the effect of our Transformer-based architecture model with the traditional CNN segmentation model, we selected the best performing models in two architectures, QueryFormer and HTC. Figure 6 shows the comparison between QueryFormer and HTC in complex scenes. The left column is the original image, the middle column is the HTC segmentation effect, and the right column is the QueryFormer segmentation effect. In the first and second scenes, the red and white boxes represent the recognition effect of the two models on the shadow parts. In the first scene, the black shadow in the red box is actually a courtyard, whereas the HTC model recognizes the black shadow as a homestead, and QueryFormer does not make such an error. In the second scene, the shadow in the red box includes the homestead sheltered by vegetation. Although the HTC model recognizes the homestead in the shadow, it cannot correctly represent the mask. QueryFormer accurately represents the mask of the house site that is not covered by vegetation in the shadow. Some edges of a homestead in the white box are in shadow. The HTC model fails to correctly identify the edges of the homestead contained in the shadow, resulting in a missing mask, but QueryFormer avoids this problem.

### 3.4. Performance Effect and Transferability of QueryFormer

**Figure 7:**
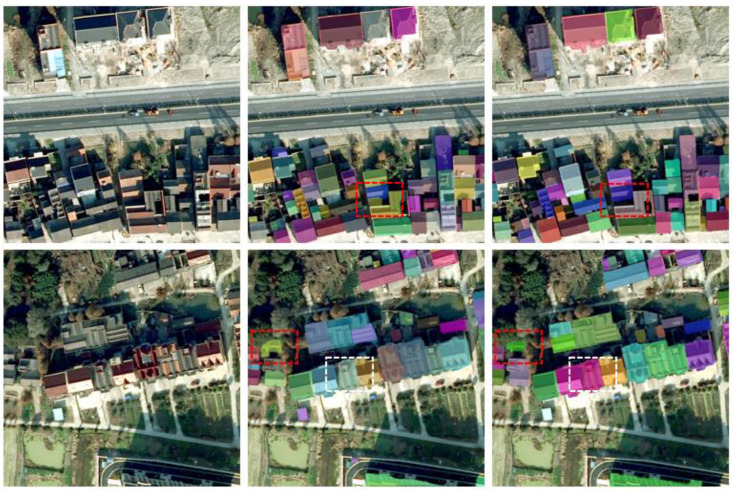
Visual comparison of the prediction results of QueryFormer and HTC. The first column is the original image, the middle column is the QueryFormer result, and the third column is the HTC result.

Figure 7 shows the effect of Swin-Transformer-backbone-based QueryFormer in a rural homestead extraction task. Whether in dense or sparse areas, QueryFormer can clearly and correctly extract the homestead. In more complex scenes, QueryFormer can precisely query each instance targets, identify objects of different sizes almost without errors, and accurately separate the background region.

**Figure 8:**
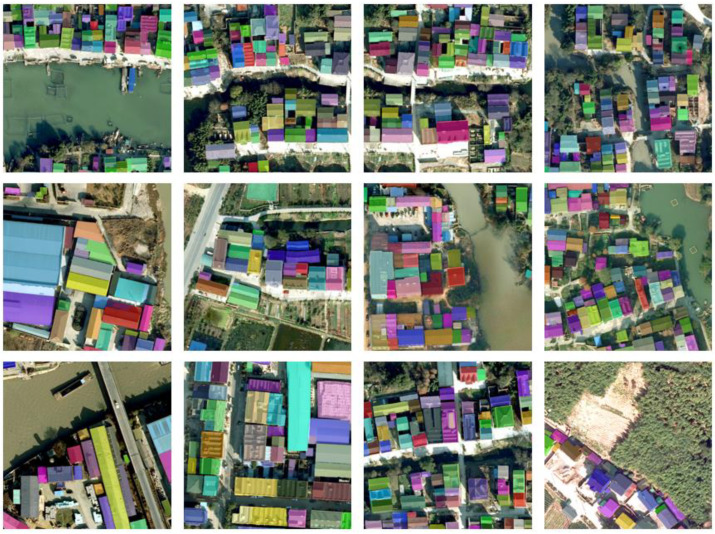
Visualization of QueryFormer instance segmentation results based on the Swin-Transformer-based backbone.

We found an interesting phenomenon in the experiment. QueryFormer also performs well on other untrained datasets. As shown in Figure 8, the first image is from a rural area, but the resolution is different from the data we trained, and the overall image is brighter due to lighting. However, QueryFormer better identified each instance of a building, but some obvious errors occurred in HTC which perform best in the CNN structure, failing to identify the buildings in the red box and yellow box. The resolution of the second image is also different from the training data, and there are some differences in roof shape and pixel distribution. QueryFormer is good at identifying most targets, but HTC misses identifying many buildings, such as foreground targets in red and yellow boxes. The third and fourth images are buildings in urban areas. There are obvious differences between building styles and buildings in rural areas, and there are many unknown distributions in the background pixels. It is difficult to transfer from rural areas to urban area. For example, in the fourth image, QueryFormer misidentifies a road as a target in a light green box, but HTC does not. However, in other areas, such as red and yellow boxes, HTC has obvious recognition errors, and QueryFormer can correctly identify most of the targets. The comparison results show the advantages of our Transformer-based architecture in transferability. The foreground features are highlighted by establishing local and global context relations. When the building texture, shape, and brightness distribution are different from the training data, the object type can also be inferred through the context relations, and then each target region can be obtained by querying.

### 3.5. Hyperparameters Contrast Experiment

In the QueryFormer model structure, some hyperparameters settings play a key role, which directly determines the effect of the model. In order to get the best hyperparameters, we set up several ablation experiments to verify the most appropriate choice of hyperparameters. In the ablation experiment, we used Swin-Transformer as the backbone and trained 12 epochs without special instructions.

**The effectiveness of the query quantity.** In order to compare the influence of different query numbers in the Transformer decoder on the results, we selected four query numbers for the comparative experiment (100–400). Please note that the queries number here are the quantity of queries in each group; we use five groups for training and one group for inferencing. As shown in Table 2, the more queries there are, the better effect is, but the calculating costs also increase substantially. For example, when we calculate the self-attention, the product shape of q and k is N×N, and N is the number of queries. When N increases from 300 to 400, N×N increases by 10,000, which brings a lot of computing overheads. In addition, after the number of queries reaches to 300, the effect does not increase significantly. When the number of queries is 400, each evaluation index has only increased by 0.3, 0.2, 0, and 0.4. Considering the effect and calculation cost, we chose 300 queries for the experiment.

**The effectiveness of the group quantity.** To compare the impact of the number of grouped queries on the results, we set five groups number for comparative experiment (1, 2, 3, 4, 5). When the group number equals 1, it is the same as the original DETR. We only use the first set of queries in the inference process. As shown in Table 3, the more groups there are, the better effect is. The model trained with group query obtains 2.7 mAP and 2.0 mAP gains over the baseline under the 12 epochs and 50 epochs. However, in fact, the calculating costs are also increasing a lot in training stage. When the groups number increases from 1 to 5, the total queries number increases from 300 to 1500. Although the inference stage does not increase the calculation cost, it will bring additional costs in the training stage. Considering that increasing the number of groups does not bring a significant rise point, the number of groups selected in our experiment is five. Moreover, during training for 50 epochs, the mean average precision (mAP) is observed to be 2% lower when the number of groups is 1 (i.e., the original DETR decoder) as compared to when the number of groups is 5. This suggests that grouping queries can enhance accuracy and expedite model training.

## 4. Discussion

### 4.1. What Deformable Attention Learns in QueryFormer Encoder

**Figure 9:**
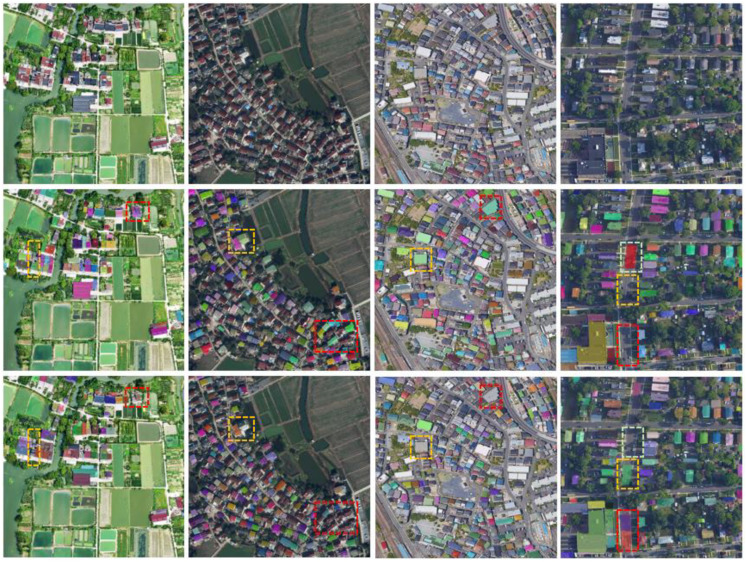
QueryFormer and HTC are tested on other untrained data. The first line is the original image, the second line is the QueryFormer result, and the third line is the HTC result.

**Figure 10:**
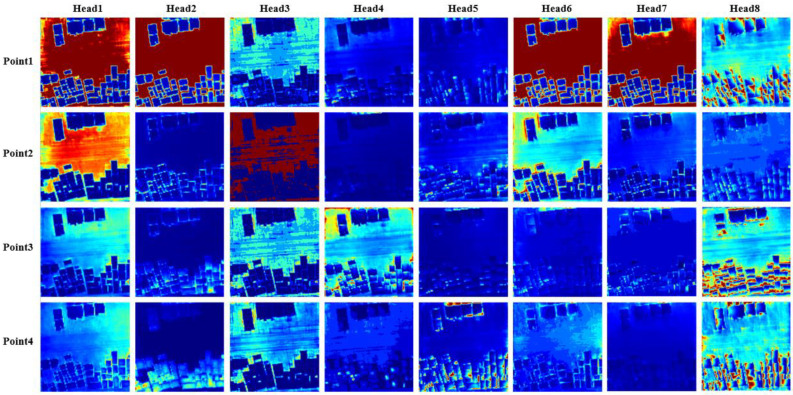
Deformable attention visual analysis of the highest resolution feature map (8×). Each column represents each head feature, and each row represents each offset sampling point feature.

Deformable attention module saves a lot of network training consumption and helps the network focus on the interest region faster. We visualize what each sample point of each attention head learns when the sample point is 4 in Figure 9 and Figure 10. Figure 9 and Figure 10 are heat maps of the attention distribution of highest resolution (8×) and lowest resolution (32×), respectively. The highlighted part of the color shows that the higher the attention weight, the more attention is paid to this region. The attention distribution deviation at high resolution is significant, and the basic contour of the target can be seen through the attention distribution, whereas the attention features extracted at low resolution are more abstract. In order to better visualize the results, we superimpose the attention map at low resolution and the original image.

In Figure 9, we observed that most of the points pay more attention to the details of the image and have higher weight on the target contour of the homestead object, such as the offset points corresponding to the highlighted parts of the homestead contour in head2, head5, head6, head7, and head8. We find that head8 also pays attention to the contour information in all directions of the homestead. Some points focus on the background information of the image, such as point1 in head1, head2, head6, and head7. In Figure 10, most of the points pay more attention to the advanced information of the image and have higher weight on the semantic part of the homestead, such as the points in head2, head4, head5, head7, and head8, which highlight the homestead object part. At the same time, some points focus on the background information of the image, such as point1 in head1, and head2. It is worth noting that the distribution of the attention weights of point2 and point3 of head3 in the attention heat map of the lowest resolution is somewhat special. We think it is a response to the context relationship between some homestead pixels and background pixels.

### 4.2. What Query Learns in QueryFormer Decoder

**Figure 11:**
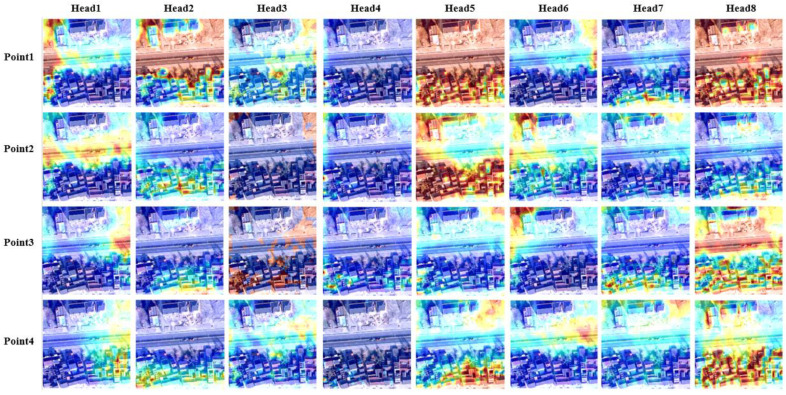
Deformable attention visual analysis of the lowest resolution feature map (32×). Each column represents each head feature, and each row represents each offset sampling point feature.

Similar to the region proposal proposed by Faster-RCNN, learnable query in the cross-attention module of the Transformer decoder learns the masks of the interest region proposals from the feature map in the encoder and gives higher weight to the target reg ion. To explore what features query has learned in the encoder, we selected the first 15 of the 300 queries in the first group for visualization experiments in Figure 11. We observe that the weights of the first 15 queries are distributed in different regions, corresponding to different homestead target objects, and the contour of the highlighted part of the attention is a complete homestead, which shows that each learnable query has completely queried the foreground target information of different regions, which can be used as the region proposal masks.

### 4.3. What Group Queries Learns in QueryFormer Decoder

**Figure 12:**
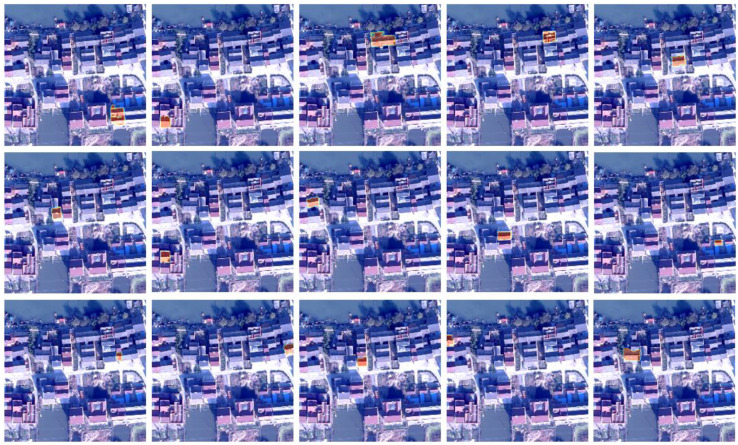
Learnable queries visual analysis of the highest resolution feature map (8×). Each column represents each head feature, and each row represents each offset sampling point feature.

**Figure 13:**
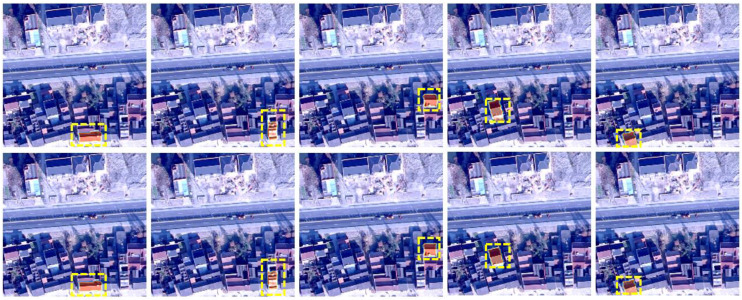
Group queries visual analysis of the highest resolution feature map (8×). Each column represents each head feature, and each row represents each offset sampling point feature.

Similar to the strategy of anchor-based detection algorithms, such as Faster RCNN in sample assignment, group queries assignment multiple positive objects to the same GT, and multiple parallel queries can quickly find the foreground region of interest. To investigate the learning outcomes of various groups’ queries, we chose the first group’s queries and one from among them. Then, we examined the attention distribution of the corresponding queries in the two groups. As shown in Figure 12, the highlighted part represents the place with high attention weight, and the yellow box is the feature area extracted from the Nth query of the two groups. By comparison, as shown in Figure 13, it is found that the region features learned by the query at the Nth position of the two groups are consistent, indicating that the information of multiple positive sample label allocation matching is consistent. In the reasoning stage, only the query features of the first group are required, and no subsequent NMS operations are required. 

## 5. Conclusions

In this study, we propose a completely end-to-end automatic instance segmentation network QueryFormer based on Transformer architecture, which can provide intelligent decision analysis of rural homesteads. QueryFormer combines the advantages of DETR. Instead of using the current mainstream instance segmentation network idea of first detection and then segmentation, it uses multiple groups of embedded vectors to directly query the characteristics of the current region. The encoder of QueryFormer uses multi-scale deformable attention module instead of the traditional self-attention module, so that the network can learn more representative sampling points, which greatly reduces the computing cost. In order to solve the characteristics of slow and long training times of the DETR-based network, multiple groups of queries are used for parallel computing in the decoder, and the one-to-one positive sample assignment calculation similar to the DETR is performed in each group. In each group, a GT is assigned to a query, and when there are K groups, a GT can be calculated by K queries. Then, K queries are used to learn the characteristics of the region from the feature maps in the encoder, and generates K proposal regions. In the inference process, the calculation will not be increased, and only the first group of queries need to be use for generating predictions. The experiment shows that our QueryFormer achieves the best performance with many state-of-the-art instance segmentation models, and when using multiple groups query to train 50 epochs, the mAP, AP_S_, AP_M_, and AP_L_ of our model are 52.8, 29.9, 58.6, and 64.7, respectively. Our method has been verified to have the best results and better transferability among various evaluation indicators, and has been verified to have faster convergence speed and better effect when using group queries. It can be well applied to the extraction of rural homestead in dense regions and be used for subsequent accurate quantitative analysis and visualization.

## Figures and Tables

**Table 1:** Quantitative evaluation (%) of several SOTA algorithms with different backbones on remote sensing images in the test set. Values in bold are the best.

Method	Backbone	Query Method	AP	AP_S_	AP_M_	AP_L_
Mask-RCNN	R101	Anchors	39.8	23.8	48.3	51.1
Cascade-RCNN	R101	Anchors	44.5	25.9	51.8	53.5
HTC	R101	Anchors	47.1	**28.6**	53.3	59.9
QueryInst	R101	300 Queries	46.5	28.2	54.1	60.2
Mask2Former	R101	300 Queries	47.7	27.4	54.8	60.8
QueryFormer	R101	300 Queries	**48.3**	27.9	**55.7**	**61.6**
Mask-RCNN	Swin-B	Anchors	44.2	27.1	54.1	59.4
Cascade-RCNN	Swin-B	Anchors	46.7	27.9	55.7	61.4
HTC	Swin-B	Anchors	51.1	**30.4**	56.5	62.3
QueryInst	Swin-B	300 Queries	50.3	30.1	56.9	62.5
Mask2Former	Swin-B	300 Queries	52.0	29.3	57.6	63.8
QueryFormer	Swin-B	300 Queries	**52.8**	29.9	**58.6**	**64.7**

**Table 2:** Comparative experimental results of query quantity.

Model	Queries	Schedules	AP	AP_S_	AP_M_	AP_L_
QueryFormer	100	12e	37.6	16.1	44.7	47.3
QueryFormer	200	12e	39.2	18.9	47.8	52.0
QueryFormer	300	12e	41.4	20.6	49.5	53.7
QueryFormer	400	12e	41.7	20.8	49.5	54.1

**Table 3:** Comparative experimental results of group quantity.

Model	Groups	Schedules	AP	AP_S_	AP_M_	AP_L_
QueryFormer	1	12e	38.7	18.4	46.1	49.7
QueryFormer	2	12e	40.5	19.4	47.8	51.3
QueryFormer	3	12e	40.8	19.9	48.4	52.4
QueryFormer	4	12e	41.1	20.3	49.1	53.2
QueryFormer	5	12e	41.4	20.6	49.5	53.7
QueryFormer	1	50e	50.8	27.1	55.4	61.9
QueryFormer	5	50e	52.8	29.9	58.6	64.7

## Data Availability

The data and code for classification are available at the following GitHub repository: https://github.com/wr19960001/QueryFormer (accessed on 30 December 2022).
